# Strong Twirling-Rotating Manual Acupuncture with 4 r/s Is Superior to 2 r/s in Relieving Pain by Activating C-Fibers in Rat Models of CFA-Induced Pain

**DOI:** 10.1155/2021/5528780

**Published:** 2021-10-12

**Authors:** Simin Song, Yuan Xu, Jiang Liu, Yadi Jia, Xiaowei Lin, YangYang Liu, Yi Guo, Hong Wang, Yongming Guo

**Affiliations:** ^1^Research Center of Experimental Acupuncture Science, Tianjin University of Traditional Chinese Medicine, Tianjin 301617, China; ^2^College of Traditional Chinese Medicine, Tianjin University of Traditional Chinese Medicine, Tianjin 301617, China; ^3^College of Acupuncture and Massage, Tianjin University of Traditional Chinese Medicine, Tianjin 301617, China

## Abstract

**Background:**

Manual acupuncture (MA) with different stimulus frequencies may give rise to varying acupuncture effects. However, the intensity-effect relationship and the underlying mechanisms of MA remain unclear.

**Objective:**

To compare the analgesic effects of different frequencies of twirling-rotating MA on rats with complete Freund's adjuvant- (CFA-) induced pain and explore the underlying mechanism via peripheral sensory nerves.

**Methods:**

First, 36 healthy male Wistar rats were randomly divided into 6 groups: control group, 2 r/s MA group (twirling-rotating MA with the frequency of 2 revolutions per second), 4 r/s MA group (twirling-rotating MA with the frequency of 4 revolutions per second), CFA group, CFA + 2 r/s MA group, and CFA + 4 r/s MA group. Rats in three CFA groups received an intraplantar injection of CFA to establish a pain model, while the rats in other three groups received an intraplantar injection of saline. Rats in the 2 r/s MA group and 4 r/s MA group were treated with the corresponding frequencies of twirling-rotating MA on bilateral Zusanli (ST36) and Kunlun (BL60) for 7 days. The ipsilateral nociceptive thresholds (paw withdrawal latency; PWL) were tested to evaluate the analgesic effects. Second, 9 healthy male Wistar rats were randomly divided into 3 groups: control group, 2 r/s MA group, and 4 r/s MA group. The proportion of C-fiber neurons (calcitonin gene-related peptide- (CGRP-) positive neurons) and A-fiber neurons (neurofilament 200- (NF200-) positive neurons) in the dorsal root ganglia (DRG) activated by MA were quantitatively analyzed with the morphological immunofluorescence staining method. Third, 30 healthy male Wistar rats were randomly divided into 6 groups: control group, CFA group, CFA + 2 r/s MA group, CFA + 2 r/s MA + RTX group, CFA + 4 r/s MA group, and CFA  + 4 r/s MA + RTX group. Resiniferatoxin (RTX) was injected into the acupoints before acupuncture. PWL was evaluated to investigate the analgesic effect.

**Results:**

Both types of MA treatment increased the PWL of saline-injecting rats and pain model rats. Moreover, 4 r/s MA was superior to 2 r/s MA in increasing PWL. A higher quantity of excited C-fiber neurons was observed following 4 r/s MA than 2 r/s MA, while the reverse was observed in the activation of A-fiber neurons. Following the injection of RTX to inhibit the activation of C-fibers, the analgesic effect of 4 r/s MA reduced significantly but not of 2 r/s MA.

**Conclusion:**

Strong MA (4 r/s MA) has superior analgesic effects to gentle MA (2 r/s MA) on CFA model rats, which is associated with C-fiber activation.

## 1. Introduction

Acupuncture is an invaluable heritage of Chinese medicine that is being practiced in 183 countries for treating diseases [1]. In recent years, several high-quality clinical studies have demonstrated the therapeutic effect of acupuncture [2–4]. The acupuncture effectiveness is influenced by many factors, such as the selection of acupuncture points and application of acupuncture modalities, including electroacupuncture (EA) [5], manual acupuncture (MA) [6], and laser acupuncture (LA) [7, 8]. For each acupuncture modality, different parameters or intensities also affect the acupuncture effect. MA is wildly used in clinical practice, which involves inserting a disposable, sterile needle to the acupuncture point (acupoint), and manipulating the needle with manual operations, such as lifting-thrusting and twirling-rotating needle in order to elicit Deqi sensation and to produce a therapeutic effect. Variations in the frequency, forcefulness, amplitude, and duration of the operations may produce a different stimulation intensity that affects the effectiveness of acupuncture treatment [9–13]. However, the intensity-effect relationship of MA and its mechanism remains unclear.

The acupuncture-induced analgesic effect has been valuable in alleviating diverse pains, including headache, muscle pain, joint pain, visceral pain, and postoperative pain. Pain disease is the main indication for the application of acupuncture. In EA, it was reported that strong current intensity is superior to weak current intensity in inducing the analgesic effect [14], However, the intensity-analgesic effect relationship in MA requires further investigation. MA manipulation can activate variable types of primary sensory afferent nerve fibers, including A-fibers and C-fibers [15, 16]. It is unclear if the types and degrees of nerve fibers activated by acupuncture are affected by the diverse frequency of MA and whether the nerve fibers mediating the analgesic effect demands further study. In this study, we postulated that different frequencies of MA resulted in variable analgesic effects, and the underlying mechanism was related to the types and degrees of activated primary sensory afferent nerve fibers. Rats with pain induced by complete Freund's adjuvant (CFA) were used to compare the analgesic effect produced by two frequencies of twirling-rotating MA. The primary sensory neurons in the dorsal root ganglia (DRG) were examined to elucidate the discrepancy in types and degrees of activated primary sensory nerve by different MA frequencies. Resiniferatoxin (RTX) was used to explore whether C-fibers mediated the analgesic effect of two frequencies of twirling-rotating MA.

## 2. Materials and Methods

### 2.1. Animals Preparations

A total of 75 healthy Wistar rats (200–220 g, male, 6–8-week-old) provided by the Beijing Weitong Lihua Company, license number: SCXK (Beijing, 2016-0006) were used in this study. Experimental procedures were carried out in accordance with the Guidance Suggestions for the Care and Use of Laboratory Animals of the Ministry of Science and Technology of China and were approved by the Animal Ethics Committee of Tianjin University of Traditional Chinese Medicine in China (TCM-LAEC2012009). The rats were reared in an environment of 12:12 hours alternating light/dark cycle, with a temperature of 25 ± 1°C, relative humidity of 50 ± 5%, and had free access to food and water.

### 2.2. Experimental Procedures

**Figure 1:**
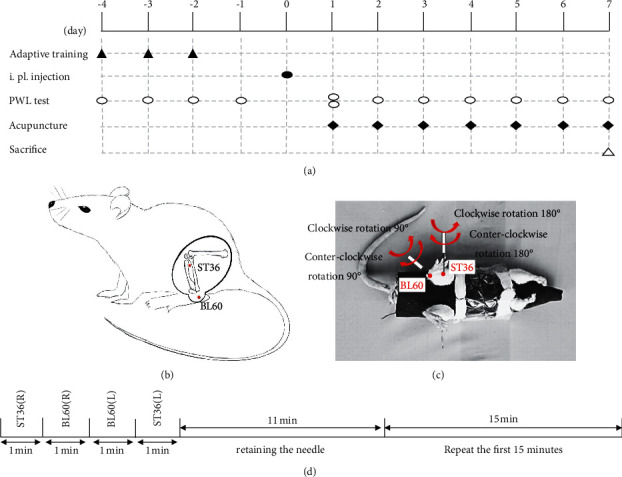
The diagram detailing the scheduled experiments and details of MA treatment. (a) The diagram of daily scheduled experiments. Adaptive training was conducted from day −4 to day −2. The CFA model was established on day 0. The PWL tests were conducted on day −4 to −1, day 1 (before and after MA), and day 2 to day 7 (after MA). MA was performed on days 1–7. Rats were sacrificed on day 7. (b) Locations of acupoints of ST36 and BL60 used in this study. (c) Rats were fixed with self-made wearing, the fixation, and the twirling direction and angle of MA on each acupoint. (d) Timeline of MA manipulation.

The study consisted of three experiments. First, 36 healthy male Wistar rats were randomly divided into 6 groups: control, 2 r/s MA, 4 r/s MA, CFA, CFA + 2 r/s MA, and CFA + 4 r/s MA groups. The duration of the experiment was 12 days.  Figure 1(a) illustrates the procedures. The rats received adaptive training of nociceptive threshold (paw withdrawal latency; PWL) testing during the first three days (day −4 to −2) and then received the formal nociceptive threshold testing as the basic value before modeling (day −1). On day 0, rats in the CFA groups received left intraplantar (i.pl.) injection of CFA to establish a pain model, while rats in the other three groups received a saline injection. MA treatments were applied to rats in the two 2 r/s MA groups and two 4 r/s MA groups on the day after CFA/saline injection, once a day, for 7 days (day 1 to 7). The PWL testing was carried out on days 1–7 (once a day, 30 min after acupuncture) to evaluate the analgesic effects using different twirling frequencies of MA. Two PWL testings were performed on day 1, once before acupuncture as the value after modeling and once after acupuncture, which was the value for the first treatment. Rats were sacrificed on day 7 after PWL testing.

Second, the types and degrees of primary sensory afferent neurons activated by different MA were examined. A total of 9 healthy male Wistar rats were randomly assigned into 3 groups: control, 2 r/s MA, and 4 r/s MA groups. The proportion of C-fiber neurons (CGRP-positive neurons) and A-fiber neurons (NF200-positive neurons) in the DRG activated (colabeled with C-FOS) by MA were quantitatively analyzed with the morphological immunofluorescence staining method to ascertain the activated DRG neurons influenced by different frequencies of MA. The rats in the two MA groups received acupuncture once and then sacrificed for the collection of tissue samples.

The third experiment was conducted to ascertain if the inhibition of C-fiber affected the analgesic effect of the two forms of MA. A total of 30 healthy male Wistar rats were randomly assigned into 6 groups: control, CFA, CFA + 2 r/s MA, CFA + 2 r/s MA + RTX, CFA + 4 r/s MA, and CFA + 4 r/s MA + RTX groups. Since the vehicle of RTX had no effect on the pain threshold of CFA rats (Figure S1, Supplementary file), no vehicle group was arranged in this experiment. The rats except for the control group received intraplantar CFA to establish a pain model. PWL was evaluated to determine the analgesic effect of acupuncture after injecting the RTX to the corresponding acupoints, and the duration of the third experiment was the same as the first experiment.

### 2.3. Pain Model

Pain models were constructed by injecting 0.1 ml CFA (every 1 ml contained 1 mg heat-killed and dried *Mycobacterium tuberculosis*, 0.85 ml paraffin oil, and 0.15 ml mannitol monooleate) (Sigma, San Diego, CA, USA) subcutaneously to the right sole of the rats. The model was successfully established after injection of CFA when the pain threshold of rats appeared significantly lower than that of the control group. The whole procedure was performed with strict sterility.

### 2.4. Acupuncture

Rats were fixed with a soft cloth to ensure a smooth process during acupuncture. Acupuncture needles (0.30 mm × 25 mm, Hanyi TCM, Beijing, China) were inserted vertically into ST36 and BL60 bilaterally with a depth of 5 mm and 3 mm, respectively. ST36 was located 3-4 mm below and 1-2 mm lateral to the midline of the knee joint [17], while BL60 was located in the depression between the prominence of the external malleolus and the Achilles tendon, posterior to the external malleolus [18], as shown in Figure 1(b). After inducing the Deqi sensation (the operator felt tight under the needle), two forms of the frequency of twirling-rotating MA were performed on ST36 and BL60. Rats in the 2 r/s MA group were treated with twirling-rotating MA with a twirling speed of 2 revolutions per second (2 r/s) bidirectionally, and rats in the 4 r/s group received twirling-rotating MA with a twirling speed of 4 revolutions per second (4 r/s) bidirectionally. In this study, one revolution of twirling-rotating MA referred to 180° bidirectional rotation in the ST36 and 90° bidirectional rotation in the BL60, as shown in Figure 1(c). Each acupoint was stimulated with twirling manipulation for 1 min and then retained the needle for 11 mins. The twirling procedure was repeated with a total acupuncture time of 30 mins (Figure 1(d)). The rats were acupunctured once a day from day 1 to day 7 after modeling, while the control and CFA groups were only immobilized and fixed without intervention. To ensure the consistency and reproducibility of the depths, amplitudes, and frequencies of twirling-rotating MA, the acupuncturist was instructed to practice two forms of MA manipulation on the ATP-II acupuncture manipulation parameter tester (manufactured by the Shanghai University of Traditional Chinese Medicine, Shang Xin Medical Technology Company). The practicality and assessment of MA were described previously by Gao et al. [19].

### 2.5. Measurement of Nociceptive Thresholds

PWL of rats was used as an indicator of the nociceptive threshold. This was measured using a thermal pain stimulator (BME-410C, Institute of Biomedical Engineering, Chinese Academy of Medical Sciences, Tianjin, China). Before the measurement, rats were placed into a plastic box (22 cm × 11 cm × 28 cm) with a glass plate underneath for 30 mins to adapt to the measurement environment. A high-intensity light bulb producing thermal radiation was applied to the fixed position of the right hind paw from underneath the glass floor. Thermal PWL was then measured and recorded. For baseline latencies, the intensity of thermal radiation was adjusted to obtain a PWL of 16–20 s in the noninflamed paw (before CFA). If the PWL reached 30 s, the thermal radiation would be halted to prevent tissue damage. The required time (s) until paw withdrawal indicated the thermal nociceptive threshold. The PWL in response to thermal stimuli was measured in triplicate with 5 mins interval.

### 2.6. Immunofluorescence Staining

Rats were anesthetized by intraperitoneal injection of 10% pentobarbital sodium (0.30 ml/100 g) and perfused intracardially with phosphate-buffered saline, followed by 4% paraformaldehyde at a rate of 20 ml/min. The left L4 DRG was removed, postfixed in 4% paraformaldehyde solution for 48 h, immersed in 30% sucrose solution to dehydrate, embedded in an optimal cutting temperature compound, and cryosectioned into 4 um slices using the Cryotome E (Thermo, USA). The primary antibodies were C-FOS antibody (1:200, EL900233, EterLife, UK), NF200 antibody (1:200, EL900264, EterLife, UK), and CGRP antibody (1:200, EL901165, EterLife, UK). The secondary antibody was a mixture of Cy3 goat anti-rabbit (1:300, EL990001, EterLife, UK) and Alexa Fluor 488 goat anti-mouse (1:400, EL990002, EterLife, UK). DAPI (1:500, G1012, Biosnail) was used for nuclear staining. After antigen retrieval using a microwave, sections were blocked in 3.0% BSA at 37°C for 30 mins and incubated with the primary antibody at 4°C in a humidor. After overnight incubation, DRG sections were washed in PBS for 15 mins and incubated with the secondary antibody at 37°C for 50 mins in a dark condition and observed under the Nikon Eclipse C1 fluorescence microscope (Cy3 red excitation wavelength 510–560, emission wavelength 590 nm, Alexa Fluor 488 green excitation wavelength 465–495, emission wavelength 515–555 nm, DAPI blue excitation wavelength 330–380, and emission wavelength 420 nm) (Nikon, Tokyo, Japan). Colabeled cells were analyzed with 3DHistech and CaseViewer Modules 2.0 RTM software version v2.0.2.61392. Through this, the semiquantitative analysis was performed by computing the percentage of cells in which the two makers colocalized, and the number of cells is labeled per field with a single marker.

### 2.7. RTX Configuration and Injection

Resiniferatoxin (RTX) powder (Absin, Shanghai) was dissolved in 100% ethanol, further diluted to 100  ug per 1 mL in a vehicle consisting of 10% Tween-80 and 10% anhydrous ethanol in normal saline, and stored at −80°C. RTX for injection was freshly prepared by diluting to 0.1 ng/ul in a vehicle and brought to room temperature prior to injection. RTX (50 ul per acupoint) was injected into the ST36 and BL60 acupoints bilaterally before acupuncture, while control rats were treated with injections of 50 *μ*L of the vehicle into ST36 and BL60 points bilaterally.

### 2.8. Statistical Analysis

Statistical analyses were performed by using the SPSS 20.0 software. All data were expressed as mean ± standard error of the mean. Multiple measurements at each different time point of nociceptive thresholds were analyzed by two-way ANOVA. One-way ANOVA for independent variables was used to compare the differences in DRG cells among the groups. The LSD method was used if accorded with the homogeneity test of variance; otherwise, Dunnett's T3 method was used. A *p* value of <0.05 was considered statistically significant.

## 3. Results

### 3.1. The Analgesic Effects of Twirling-Rotating MA with Different Frequencies

The PWL in the control group did not change significantly over time, which indicated that intraplantar injection of saline on healthy rats had no effect on PWL.

In the 2 r/s MA group, PWL was increased on days 1–7 when compared with the control group, with a significant difference on day 2 (21.51 ± 0.44 s vs. 18.25 ± 1.39 s, *P* < 0.05), day 3 (20.34 ± 0.20 s vs. 18.41 ± 0.94 s, *P* < 0.05), day 4 (20.55 ± 0.69 s vs. 18.01 ± 1.05 s, *P* < 0.05), day 5 (22.17 ± 0.74 s vs. 17.62 ± 0.93 s, *P* < 0.01), day 6 (22.56 ± 0.19 s vs. 18.64 ± 1.35 s, *P* < 0.01), and day 7 (20.82 ± 0.52 s vs. 18.73 ± 0.93 s, *P* < 0.05).

In the 4 r/s MA group, PWL was also increased on days 1–7 when compared with the control group, with significant difference showed on day 1 (23.84 ± 0.99 s vs. 18.82 ± 1.09 s, *P* < 0.01), day 2 (23.60 ± 0.73 s vs. 18.25 ± 1.39 s, *P* < 0.01), day 3 (22.31 ± 0.49 s vs. 18.41 ± 0.94 s, *P* < 0.01), day 4 (22.94 ± 0.69 s vs. 18.01 ± 1.05 s, *P* < 0.01), day 5 (23.99 ± 0.68 s vs. 17.62 ± 0.93 s, *P* < 0.01), day 6 (24.92 ± 0.53 s vs. 18.64 ± 1.35 s, *P* < 0.01), and day 7 (23.00 ± 0.51 s vs. 18.73 ± 0.93 s, *P* < 0.01).

Besides, compared with the 2 r/s MA group, the PWL of the 4 r/s MA group were higher with a significant difference on day 1 (23.84 ± 0.99 s vs. 21.51 ± 0.44 s, *P* < 0.01), day 3 (22.31 ± 0.49 s vs. 20.34 ± 0.20 s, *P* < 0.05), day 4 (22.94 ± 0.69 s vs. 20.55 ± 0.69 s, *P* < 0.05); day 6 (24.92 ± 0.53 s vs. 22.56 ± 0.19 s, *P* < 0.05), and day 7 (23.00 ± 0.51 s vs. 20.82 ± 0.52 s, *P* < 0.05). These results demonstrated that both 2 r/s and 4 r/s MA enhanced the pian threshold of healthy rats. 4 r/s MA showed a better effect in enhancing the pian threshold than 2 r/s MA.

The CFA group showed significant decrement on PWL after CFA injection, with statistical significance on days 0–7 when compared with the control group (all *P* < 0.01).

In the CFA + 2 r/s MA group, an increase in the PWL was observed on days 1–7 when compared with the CFA group, with a significant difference on day 2 (9.93 ± 0.69 s vs. 6.68 ± 0.41 s, *P* < 0.05), day 3 (9.30 ± 1.35 s vs. 6.08 ± 0.69 s, *P* < 0.05), day 6 (1.22 ± 0.57 s vs. 7.27 ± 0.86 s, *P* < 0.01), and day 7 (12.68 ± 0.40 s vs. 7.59 ± 0.99 s, *P* < 0.01).

In the CFA + 4 r/s MA group, PWL was also increased on days 1–7 when compared with the CFA group, with a significant difference demonstrated on day 1 (10.88 ± 0.91 s vs. 7.67 ± 0.85 s, *P* < 0.05), day 2 (10.54 ± 0.77 s vs. 6.68 ± 0.41 s, *P* < 0.01), day 3 (10.41 ± 1.06 s vs. 6.08 ± 0.69 s, *P* < 0.01), day 4 (10.41 ± 0.73 s vs. 7.39 ± 1.25 s, *P* < 0.05), day 5 (14.26 ± 0.95 s vs. 7.78 ± 0.65 s, *P* < 0.01), day 6 (14.17 ± 0.87 s vs. 7.27 ± 0.86 s, *P* < 0.01), and day 7(14.90 ± 0.96 s vs. 7.59 ± 0.99 s, *P* < 0.01).

**Figure 2:**
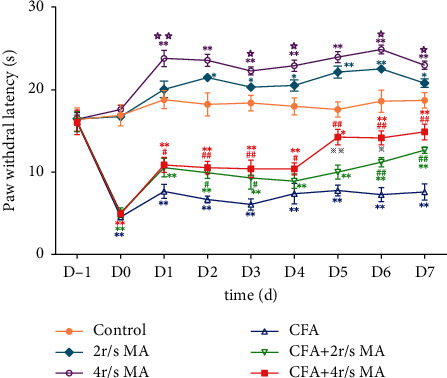
The changes of PWL affected by MA (*n* = 6). ^*∗*^*P* < 0.05 and ^*∗∗*^*P* < 0.01, compared with the control group. ^#^*P* < 0.05 and ^##^*P* < 0.01, compared with the CFA group. ^☆^*P* < 0.05 and ^☆☆^*P* < 0.01, compared with the 2 r/s MA group. ^※^*P* < 0.05 and ^※※^*P* < 0.01, compared with the CFA + 2 r/s MA group.

Furthermore, 4 r/s MA demonstrated a superior effect in increasing the PWL than 2 r/s MA. When compared with the 2 r/s MA group, the PWL of the 4 r/s MA group was higher from days 2–7 with a significant difference on day 5 (14.26 ± 0.95 s in the 4 r/s MA group vs. 10.01 ± 0.86 s in the 2 r/s MA group, *P* < 0.01) and day 6 (14.17 ± 0.87 s in the 4 r/s MA group vs. 11.22 ± 0.57 s in the 2 r/s MA group, *P* < 0.05). These results indicated that both 2 r/s and 4 r/s MA relieved pain induced by CFA, while the MA with a twirling frequency of 4 r/s demonstrated a superior analgesic effect to 2 r/s (Figure 2).

### 3.2. The Activation of Primary Sensory Neurons in the DRG by Twirling-Rotating MA with Different Frequencies

The control group only received the saline injection without MA stimulation, and the CGRP neurons and NF200 neurons were not activated significantly.

**Figure 3:**
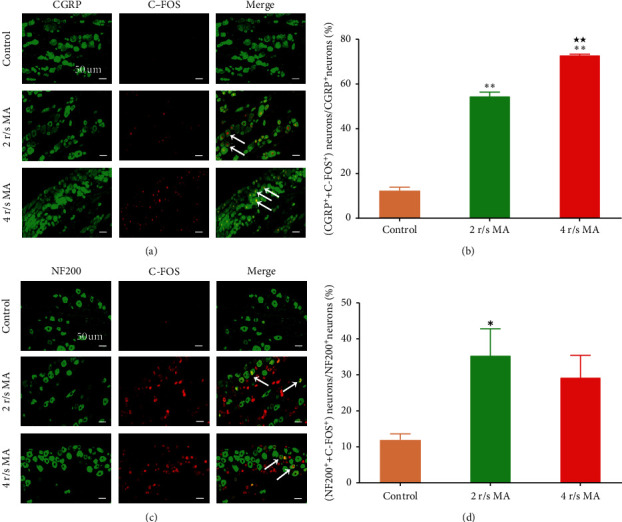
The activation of primary sensory neurons in the DRG by twirling-rotating MA with different frequencies (n = 3). (a) The representative images of C‐FOS (+) co-labeled with CGRP (+) in DRG among each group; (b) comparison of the proportion of CGRP‐positive neurons excited by twirling-rotating MA with two forms of frequencies. (c) The representative images of C‐FOS(+) co‐labeled with NF200(+) in DRG among each group; (d) comparison of the proportion of NF200‐positive neurons excited by twirling‐rotating MA with two forms of frequencies. Red represents C‐FOS; green represents NF200 or CGRP. The white arrow indicates examples of co-labeled neurons. The bar = 50 um. ^*∗*^*P* < 0.05; ^*∗∗*^*P* < 0.01, compared with the control group; ^★^*P* < 0.05; ^★★^*P* < 0.01, compared with the 2 r/s MA group.

Compared with the control group, the CGRP neurons were activated after MA. The ratio of the activated CGRP neurons in the 2 r/s MA group and the 4 r/s MA group were 53.59 ± 1.77% and 72.93 ± 0.38%, respectively, which were significantly higher than the control group (12.30 ± 1.54%, all *P* < 0.01). The difference in the ratio between the 4 r/s MA group and the 2 r/s MA group was significant (*P* < 0.01) (Figures 3(a) and 3(b)). These results indicated that both the strong MA (4 r/s MA) and gentle MA (2 r/s MA) activated C-fibers. Besides, the strong MA activated more C-fibers than the gentle MA.

The NF200 neurons were also activated after MA when compared with the control group. The ratio of the activated NF200 neurons in the 2 r/s MA group was 35.44 ± 7.34%, which was significantly higher than the control group (11.92 ± 1.71%, *P* < 0.05), while no significant difference was observed between the 4 r/s MA group and the control group (Figures 3(c) and 3(d)).

### 3.3. The Analgesic Effect of MA after Injecting RTX to the Acupoints

**Figure 4:**
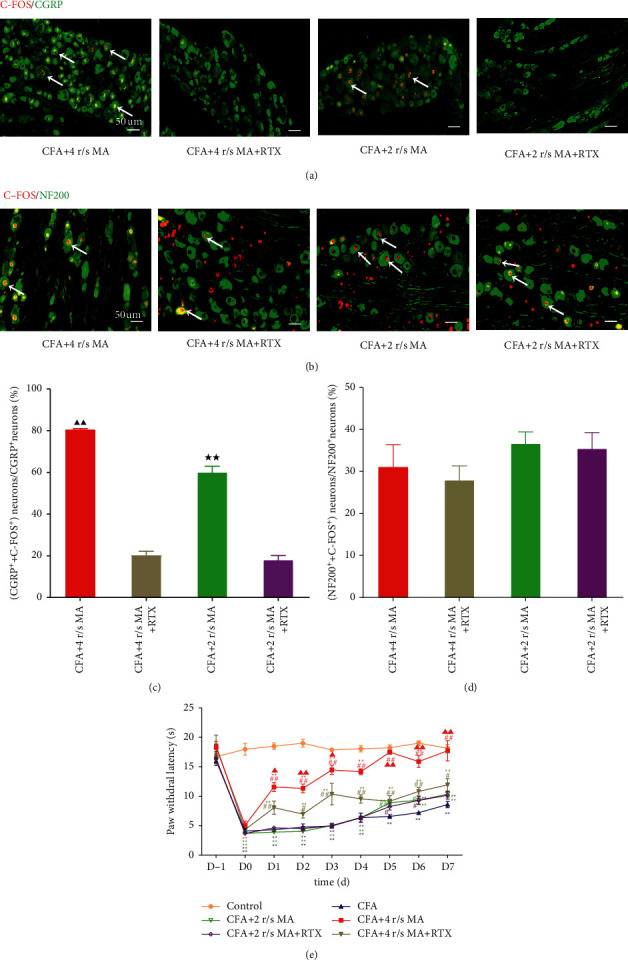
The activation of the DRG neurons and acupuncture analgesia of MA with different frequencies affected by RTX (n = 5). (a, b) The representative images of C‐FOS co-labeled with CGRP-positive neurons and C‐FOS co‐labeled with NF200‐positive neurons in the DRG among each group. The white arrows indicated examples of co-labeled neurons. Bar = 50 um. (c, d) Comparison of the proportion of CGRP-positive neurons and NF200‐positive neurons between the 4 r/s MA, 4 r/s MA+ RTX, 2 r/s MA, 2 r/s MA + RTX, and CFA groups. (e). The analgesic effects of 2 forms of MA affected by RTX. The white arrow indicates examples of co-labeled neurons. ^*⋆*^*P* < 0.05; ^*⋆⋆*^*P* < 0.01, compared with the control group; ^*#*^*P* < 0.05; ^*##*^*P* < 0.01, compared with the CFA group. ^*▲*^*P* < 0.05; ^*▲▲*^*P* < 0.01, compared with the CFA + 4 r/s MA + RTX group. ^*★★*^*P* < 0.01, compared with the CFA + 2 r/s MA + RTX group.

RTX is an ultrapotent analogue of capsaicin, which inhibits the activation of C-fiber. First, the activation of the DRG neurons between the MA group and MA + RTX group with the corresponding MA frequency was compared to ascertain the inhibition of the activation of C-fiber neurons by RTX. In the CFA + 4 r/s MA + RTX group, 20.44 ± 1.73% of the CGRP-positive neurons were activated, which was significantly less than 80.59 ± 0.40% in the CFA + 4 r/s MA group (*P* < 0.01). In the CFA + 2 r/s MA + RTX group, 18.00 ± 2.14% of the CGRP-positive neurons were activated, which was significantly less than 60.05 ± 2.93% in the CFA + 2 r/s MA group (*P* < 0.01). However, there was no significant change in the quantity of the activated NF200-positive neurons in the RTX-treated rats compared with non-RTX-treated rats (Figures 4(a)–4(d)). These findings indicated that RTX inhibited the activation of C-fiber neurons following MA, but not A-fiber neurons.

Second, we evaluated the PWL of rats to estimate the effect of RTX on acupuncture-induced analgesia. The PWL in the control group did not change significantly over time. While in the CFA group, the PWL decreased prominently with a statistical significance on days 0–7 when compared with the control group (all *P* < 0.01). Compared with the CFA group, an increase in the PWL was observed in the CFA + 4 r/s MA group with a significant difference on day 1 (4.32 ± 0.22 s vs. 11.56 ± 0.77 s, *P* < 0.01), day 2 (4.75 ± 0.56 s vs. 11.33 ± 0.74 s, *P* < 0.01); day 3 (4.95 ± 0.35 s vs. 14.46 ± 0.78 s, *P* < 0.01); day 4 (6.35 ± 0.79 s vs. 14.15 ± 0.51 s, *P* < 0.01); day 5 (6.57 ± 0.20 s vs. 17.47 ± 0.35 s, *P* < 0.01); day 6 (7.20 ± 0.19 s vs. 15.88 ± 0.98 s, *P* < 0.01); and day 7 (8.59 ± 0.53 s vs. 17.70 ± 1.74 s, *P* < 0.01). Compared with the CFA + 4 r/s MA group, a decrease in the PWL was observed in the 4 r/s MA + RTX group with a significant difference on day 1 (11.56 ± 0.77 s vs. 8.06 ± 1.13 s, *P* < 0.05); day 2 (11.33 ± 0.74 s vs. 6.96 ± 0.69 s, *P* < 0.01); day 3 (14.46 ± 0.78 s vs. 10.36 ± 1.83 s, *P* < 0.05); day 5 (17.47 ± 0.35 s vs. 9.16 ± 0.91 s, *P* < 0.01); day 6 (15.88 ± 0.98 s vs. 10.81 ± 0.65 s, *P* < 0.01); day 7 (17.70 ± 1.74 s vs. 11.93 ± 1.08 s, *P* < 0.01). In addition, compared with the control group, the PWL in the CFA + 2 r/s MA group was increased significantly on day 5 (6.57 ± 0.20 s vs. 8.90 ± 0.34 s, *P* < 0.05) and day 6 (7.20 ± 0.19 s vs. 9.32 ± 0.64 s, *P* < 0.05). There was no significant difference between the CFA + 2 r/s MA group and the CFA + 2 r/s MA + RTX group (Figure 4(e)). These results indicated that C-fiber participated in the analgesic effect of 4 r/s MA but not of 2 r/s MA.

## 4. Discussion

In the present study using the CFA-induced pain rat model, the analgesic effects of MA with two forms of twirling frequency were explored. Our findings revealed an increase in the PWL by the two MA in rats receiving CFA injection, which was consistent with the studies by Zheng et al. [20] and Liu et al. [21], demonstrating the analgesic effect by MA. Besides, MA can also increase the pain threshold of the healthy rats (injecting saline), which was similar with the earlier studies on healthy subjects [22, 23]. Moreover, MA with twirling frequencies of 4 r/s and 2 r/s showed varying analgesic effects. Specifically, the analgesic effect induced by MA with 4 r/s frequency was superior to 2 r/s MA, suggesting the positive “intensity (frequency)–response” correlation of MA-induced analgesia. The intensity-response relationship of MA resembles that of electroacupuncture- (EA-) induced analgesia. Lv et al. [14] have reported the superiority of strong current intensity of EA (>2 mA) to weak current intensity (<0.5 mA) in alleviating pain intensity in patients with osteoarthritis of the knee, indicating that strong stimulation is better than that of weak stimulation for inducing analgesia. In our previous study, we found that 2 r/s MA was superior to 4 r/s MA in regulating gastric motility [19], which suggested an inverse “intensity (frequency)–response” relationship as opposed to the present study. Taken together, these findings suggest that different frequencies of MA lead to variability in treatment outcomes depending on the types of disease, given that the optimal stimulation parameters of MA may vary in diverse diseases or conditions.

To explore the mechanisms of acupunctural effects induced by different twirling frequencies, we investigated the types and degrees of activated primary sensory afferent fibers induced by acupuncture. Given that the electrophysiological method is appropriate for a qualitative study of nerve fiber types but not for a quantitative study, the morphological method was used to evaluate the types and degrees of activated primary sensory neurons in DRG. Our findings revealed that both forms of twirling-rotating MA activated NF200-positive neurons (A-fiber neurons) and CGRP-positive neurons (C-fiber neurons). Indeed, several studies have shown that MA can activate both A-fiber and C-fiber [24, 25]. However, to our knowledge, the comparison of the types and degrees of peripheral afferent nerve activated by different MA frequencies has not been reported in the literature previously. Our study represented the first to demonstrate the distinct primary sensory afferent nerves activated by two forms of twirling-rotating MA with 2 r/s and 4 r/s twirling frequency. The results showed that both 2 r/s MA and 4 r/s MA activated C-fiber neurons primarily. Moreover, 4 r/s MA activated more C-fiber neurons than 2 r/s MA, which indicates that the greater the stimulation intensity, the more activated C-fiber neurons.

In this study, RTX was used to inhibit the activation of C-fiber in order to investigate the contribution of the peripheral afferent fibers to the analgesic effect following the application of twirling-rotating MA. RTX is an ultrapotent analogue of capsaicin, which activates the transient receptor potential vanilloid 1 (TRPV1) in a subpopulation of primary afferent sensory neurons that participated in nociception (the transmission of physiological pain). The activation of TRPV1 leads to the calcium influx and calcium overload, resulting in C-fiber desensitization and consequently inhibition of C-fiber activation [26, 27]. Our findings revealed that following the RTX injection in ST36 and BL60, CGRP-positive neurons were not activated upon two forms of twirling-rotating MA stimulation, while the A-fiber neurons were not affected. The analgesic effects of 4 r/s MA appeared to have decreased after RTX injection, but no significant change was observed in 2 r/s MA. These results indicated that the C-fiber mediated the analgesic effect of 4 r/s MA but not 2 r/s MA. Given that MA activates both C-fiber and A-fiber and C-fiber activation was inhibited by RTX, we speculated that the analgesic effect of 2 r/s MA may be mediated by A-fiber. Toda et al. [28] have reported a significant correlation between the amplitude of A-fibers in the compound action potentials elicited by electroacupuncture stimulation and the degree of suppression of the jaw opening reflex, suggesting a leading role of A-fibers in producing acupuncture-induced analgesia. Previous studies [29, 30] have shown that the peripheral afferent nerve fibers including both A-fiber and C-fiber contribute to analgesia by acupuncture. However, the relationship between the acupuncture parameters, the type of nerve fibers activation, and the analgesic effects remains unclear. In our study, two forms of twirling frequency of MA had been standardized to compare the analgesic effect, and we discovered differences in the analgesic effects that may be mediated by different peripheral afferent nerve fibers.

It is generally recognized that C-fiber transmits noxious stimuli that are associated with pain sensation. Our findings demonstrated that C-fiber mediated the analgesic effect of 4 r/s twirling-rotating MA, indicating that the pain induced during acupuncture inhibited the pain of CFA rats. Indeed, pain inhibiting pain has been reported in numerous studies. For instance, an experimental study showed that in patients with chronic back pain, applying acute noxious thermal stimuli to the skin of lower back induced pain relief [31]. Also, Glaucia et al. revealed that in a standard rat model of inflammatory pain following carrageenan injection into the hind paw, the subsequent capsaicin injection into the forepaw as a noxious stimulus had induced antinociception [32]. The possible mechanism for this phenomenon is that noxious stimulation triggers an ascending–descending pain modulation pathway linking the mesolimbic system to the PAG–RVM descending system [33]. In the clinical acupuncture practice, acute pain such as those caused by acute ankle sprain or acute lumbar muscle sprain can be significantly alleviated by strong stimulation of acupuncture [34–36]. Coupled with the findings from this study, acupuncture-induced analgesia can be considered as a form of pain-induced analgesia, especially MA of strong intensity. Given that MA is being performed flexibly in clinical practice by adjusting the parameters including the needling frequency, depth, and amplitude according to the patient's response, we propose the practice of gentle MA for patients with lower acupuncture tolerance to activate A-fiber for relieving pain, while a strong MA can be performed to activate C-fiber for effective pain relief in patients with higher acupuncture tolerance.

## 5. Conclusion

Twirling-rotating MA with different intensities (frequency) produce diverse analgesic effects. The analgesic effect of strong MA is superior to that of gentle MA, which is mediated differentially by peripheral afferent fibers. Strong MA activates more C-fibers that results in higher pain relief than gentle MA.

## Data Availability

The data used to support the findings of this study are available from the corresponding author upon request.

## Supplementary Materials

Supplementary Materials Figure S1. The vehicle has no significant effect on PWL of CFA rats.
