# Supplementary figures and images for: Strong Twirling-Rotating Manual Acupuncture with 4 r/s Is Superior to 2 r/s in Relieving Pain by Activating C-Fibers in Rat Models of CFA-Induced Pain
Source: Evid Based Complement Alternat Med. 2021 Oct 12;2021:5528780. doi: 10.1155/2021/5528780 (PMC8526210; doi:10.1155/2021/5528780)

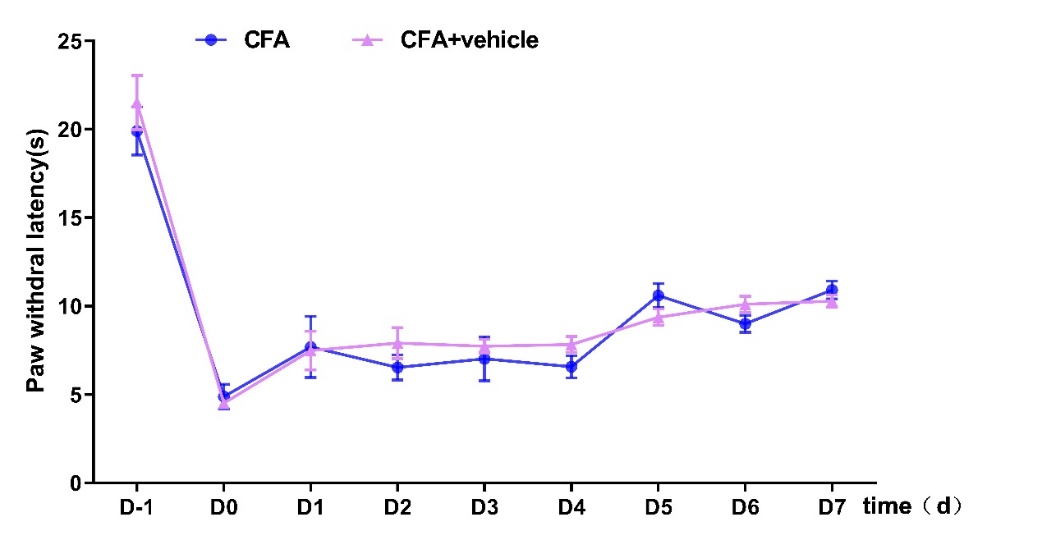


Figure S1. The vehicle has no significant effect on PWL of CFA rats.

Supplement: Supplementary Materials — Figure S1. The vehicle has no significant effect on PWL of CFA rats. [file 5528780.f1.docx]
